# Migrant workers in China need emergency psychological interventions during the COVID-19 outbreak

**DOI:** 10.1186/s12992-020-00608-w

**Published:** 2020-08-19

**Authors:** Zi-Han Liu, Yan-Jie Zhao, Yuan Feng, Qinge Zhang, Bao-Liang Zhong, Teris Cheung, Brian J. Hall, Yu-Tao Xiang

**Affiliations:** 1 Unit of Psychiatry, Institute of Translational Medicine, Faculty of Health Sciences, University of Macau, 3/F, Building E12, Avenida da Universidade, Taipa, Macau SAR, China; 2 Center for Cognition and Brain Sciences, University of Macau, Macao SAR, China; 3 Institute of Advanced Studies in Humanities and Social Sciences, University of Macau, Macao SAR, China; 4 The National Clinical Research Center for Mental Disorders & Beijing Key Laboratory of Mental Disorders, Beijing Anding Hospital & the Advanced Innovation Center for Human Brain Protection, Capital Medical University, Beijing, China; 5 Affiliated Wuhan Mental Health Center, Tongji Medical College of Huazhong University of Science & Technology, Wuhan, Hubei Province China; 6 School of Nursing, Hong Kong Polytechnic University, Hong Kong SAR, China; 7 Department of Health, Behavior and Society, Johns Hopkins Bloomberg School of Public Health, Baltimore, MD USA

**Keywords:** COVID-19, Migrant workers, Mental health, China

## Abstract

The 2019 novel coronavirus disease (COVID-19) has been found in more than 200 countries worldwide since December, 2019. In China, a major reason for the rapid transmission of the COVID-19 in early stage of the outbreak is the huge numbers of passengers boarding their “last train home” to meet family members during the Spring Festival. Most of these travelers were internal migrant workers. In order to reduce the risk of the COVID-19 transmission, public transportation networks were suspended, and many migrant workers who returned to their hometowns needed to be quarantined for 2 weeks, which led to the delay of returning back to cities to work. Many businesses have temporarily closed because of the risk of COVID-19 transmission, leading to unemployment of many workers. Sudden loss of income and further quarantine enforcement in cities can exacerbate existing mental health problems or trigger new mental disorders among affected migrant workers. However, to date no specific guidelines or strategies about mental health services of migrant workers have been released. Health authorities and professionals should pay more attention to this vulnerable group and provide timely mental health service support for those in need.

## Background

Since the 2019 novel coronavirus disease (COVID-19) was first reported in Wuhan, Hubei province, China at the end of 2019, it has been found in more than 200 countries, and gained enormous attention worldwide. In China, a major reason for the rapid transmission of the COVID-19 in early stage of the outbreak is the huge numbers of passengers boarding their “last train home” to meet family members during the Spring Festival in China. This is usually the peak season for Chinese citizens to travel to and from their hometowns. There were more than 1.14 billion trips between January 10 and January 24, 2020. Most travelers were, in fact, internal migrant workers (migrant workers hereafter) [3]. Due to mass quarantine measures and loss of income, mental health problems are common among Chinese migrant workers. A comprehensive introduction to mental health problems and related issues among migrant workers in China is warranted.

## Main text

Migrant workers refer to individuals aged 16 years and older, who leave their original residence in rural areas and work in cities for 3 months or more. Migrant workers account for around 1/5 of the whole Chinese population (approximately 300 million) [8]. On the one hand, migrant workers bring about abundant cheap labor to the cities and speed up economic growth. On the other hand, they are part of the low socio-economic population in cities. Migrant workers can only take up temporary jobs in cities because their legal residence (‘*hukou*’) is still in their hometown rural areas, rather than in the cities where they live and work. In China the “*hukou*” system is closely linked with an individual’s access to accommodation, education, social welfare, and health care. Without a “*hukou*”, migrant workers cannot be fully covered by health insurance in cities where they temporarily work. Compared to permanent residents in cities (i.e., those with ‘*Hukou*’ in cities), most migrant workers in China have insufficient financial savings, low education levels, a high level of life stress, limited time and money to see doctors or even being discriminated by others, which contributes to a higher prevalence of mental health problems than observed in the general population [10]. This claim was evidenced by a recent study which reported that the prevalence of depression was 20.1% in Chinese migrant workers [14], which was almost four times (5.9%) higher than those rural residents in China [19].

In order to reduce the risk of the COVID-19 transmission and facilitate early identification, and isolation of confirmed and suspected cases, mass quarantine was widely adopted and public transportation was suspended in many areas of China. Due to fear of infection, uncertainty of the COVID-transmission, and misinformation about the disease in social media, mental health problems, such as sleep disturbances, depression and anxiety, are common in the general public and migrant workers [4, 7, 13, 15, 17]. In addition, during the COVID-19 outbreak many migrant workers who returned to hometowns needed to be quarantined for 2 weeks. These migrants thus had to postpone their journey back to cities to work. Although roughly 1 million migrant workers were able to return to cities [16, 18], due to the potential risk of COVID-19 transmission, many businesses temporarily closed leading to unemployment. Sudden loss of income has affected their ability to support their families, and they have experienced increased discrimination, which could further exacerbate existing or trigger new mental health problems, such as boredom, anger, anxiety, and guilt, in migrant workers. However, regular mental health education is typically not available for this population during the COVID-19 outbreak, and financial hardship, further quarantine measures and stigma associated with mental illness [12] are key barriers deterring them from seeking timely mental health treatments if needed. Moreover, basic health insurance for migrant workers in many cities of China do not completely cover mental health services; furthermore, private health insurance is not affordable for migrant worker. These factors make migrant workers less likely to seek help from mental health services when needed.

In the past several months, a number of guidelines on emergency psychological interventions have been developed in China [5]. For example, the National Health Commission of China (NHC) has integrated psychological crisis intervention into general disease prevention. Major mental health associations and academic societies in China also have developed guidelines and expert consensus for mental health institutions, such as the ‘The Manual of Mental Health Services during of the COVID outbreak’. Following these guidelines, some crisis psychological services, such as 24-h hotlines and online mental health education, have been set up in many areas of China [6]. However, lack of knowledge of mental health, low education level, perceived stigma and limited access to online information make it hard for migrant workers to benefit from these services. The mental health services needs of certain special populations including older adults, children and adolescents, pregnant women, and health professionals, have been addressed in recent guidelines, such as the ‘Psychological Adjustment Guidelines for Coping with the New Coronavirus Pneumonia’ [4]. In contrast, however, the mental health of migrant workers was neglected. To the best of our knowledge, no specific guidelines on mental health of migrant workers have been released in China during COVID-19 outbreak.

Based on the currently available health resources and experiences of previous bio-disasters, several measures may be helpful to improve mental health services for migrant workers during the COVID-19 outbreak. First, relevant guidelines and/or expert consensus on mental health of migrant workers should be developed. Second, regular mental health screening should be performed for this population, such as using online self-report instruments that have recently been widely used [1, 2, 11]. Third, free and accessible mental health services, such as online psychological counseling, hotline services and other telehealth services, should be established for migrant workers. In addition, free online education on mental health is useful to improve their awareness of mental health and help them seek help from mental health services [4]. Finally, based on the experiences obtained during the Middle East Respiratory Syndrome (MERS) epidemic in Korea [9], social workers and other professionals can play an important role in providing social support and relevant education for migrant workers who are in need during the COVID-19 outbreak.

## Conclusion

Migrant workers are a vulnerable group in China during the COVID-19 outbreak. Both health authorities and health professionals should pay more attention to this population and provide a timely mental health services for those in need.

## Data Availability

Not applicable for this study.

## Abbreviation

COVID-19: The 2019 novel coronavirus disease
